# Self-Directedness and Cooperativeness, Psychosocial Dysfunction and Suffering in ESSENCE

**DOI:** 10.1155/2013/416981

**Published:** 2013-04-28

**Authors:** Danilo Garcia, Henrik Anckarsäter, Sebastian Lundström

**Affiliations:** ^1^Centre for Ethics, Law and Mental Health (CELAM), University of Gothenburg, Wallinsgatan 8, Mölndal, 431 41 Gothenburg, Sweden; ^2^Institute of Neuroscience and Physiology, The Sahlgrenska Academy, University of Gothenburg, Wallinsgatan 8, Mölndal, 431 41 Gothenburg, Sweden; ^3^Department of Clinical Sciences, Lund University, 221 00 Lund, Sweden; ^4^Swedish Prison and Probation Service, R&D Unit, Wallinsgatan 8, Mölndal, 431 41 Gothenburg, Sweden; ^5^Gillberg Neuropsychiatry Centre, Institute of Neuroscience and Physiology, University of Gothenburg, Gothenburg, 405 30 Gothenburg, Sweden

## Abstract

*Background*. The acronym ESSENCE (Early Symptomatic Syndromes Eliciting Neurodevelopmental Clinical Examinations) highlights that children seeking clinical treatment are often multiply impaired, thus requiring treatment from several specialties. The aim was to map and relate, on a population level, ESSENCE to two salient predictors of health and adaptation to adversities, namely, Self-Directedness and Cooperativeness and also to dysfunction and suffering. *Methods*. Participants were twins (*N* = 1892) aged 9 or 12 whose parents were interviewed with the Autism-Tics, ADHD and other Comorbidities inventory (A-TAC), and the Junior Temperament and Character Inventory (J-TCI). The A-TAC was first used to discern four ESSENCE-related screening diagnoses: autism spectrum disorders, attention deficit hyperactivity disorder, learning disabilities, and developmental coordination disorder; second, to quantify dysfunction and suffering in important social areas. *Results*. ESSENCE symptoms were continuously and categorically associated with deficiency in Self-Directedness and Cooperativeness and higher ratings of dysfunction and suffering. The impact of ESSENCE symptoms on these measures of mental health was found in a milder form in about 16% of all children and in a severe form in about 2%. *Conclusion*. Therapeutic interventions focusing on Self-Directedness and Cooperativeness might provide a novel method for child psychiatry in its approach to ESSENCE.

## 1. Introduction

During the last decades, the notion of mutually exclusive criteria for psychiatric disorders has been questioned [1]. Not only do mental disorders and symptoms coexist, but also share etiology. Twin studies, for example, have shown that the same etiological factors behind autism spectrums disorders (ASDs) also give rise to attention deficit hyperactivity disorder (ADHD), learning disabilities (LDs), and developmental coordination disorder (DCD) [2]. In addition, specific molecular genetic and chromosomal variants and abnormalities found in family studies of ASDs have been shown to give rise to heterogeneous arrays of clinical symptoms, corresponding to different psychiatric categorical diagnoses (e.g., learning disabilities and/or ADHD) [3, 4]. Further evidence for the lack of clear demarcations between neuropsychiatric disorders as defined in current diagnostic manuals has come from clinical and family studies, the quest for valid biomarkers, and the development of atypical neuroleptics which influence symptoms rather than diagnoses (as reviewed by [5]). Therefore, it has been concluded that coexisting disorders are, indeed, the rule rather than the exception in child psychiatry [6].

Recently, Gillberg [7] coined the acronym ESSENCE (Early Symptomatic Syndromes Eliciting Neurodevelopmental Clinical Examinations) to highlight that children seeking clinical treatment are often impaired in multiple domains and require treatment from several specialities. The ESSENCE perspective not only acknowledges the possibilities of shared aetiologies behind seemingly different conditions but also puts emphasis on cognitive problems, developmental deficits and treatment opportunities that are similar across diagnostic categories. Hence, advocating broad clinical assessments, and avoidance of compartmentalisation into specific diagnoses and “disease-specific clinics.” In addition it highlights the understanding of diagnostic shifts (i.e., language impairments to ASDs, or ADHD to ASDs) [8, 9] and also that children with ESSENCE conditions would benefit from a wide array of treatment possibilities that include, but are not limited to, pediatricians, social workers, language therapists, child neurologists, psychologists, and geneticists.

The named conditions of ESSENCE were initially thought of as discrete categories (a child either had or did not have ASDs or ADHD), but population-based studies have invariably shown that the symptoms thought to identify these conditions are dimensionally distributed in the general population without “zones of rarity.” In addition, recent studies have failed to identify any etiological demarcations between autistic-like traits and ASDs [10] or ADHD-related traits and ADHD [11]. The distribution of traits varies; few children have, for example, conduct problems while a majority have had some ADHD problem, at least “to some degree,” at some stage of their lives [12].

The named conditions of ESSENCE have theoretical and clinical links with personality disorders in adulthood; Asperger's disorder was initially described as a form of schizoid personality disorder in children [13], conduct disorder is by definition a prelude to antisocial personality disorder [14], anorexia nervosa has been linked to anancastic and alexithymic personalities, and longitudinal studies have shown that ADHD carries an increased risk for antisocial personality disorder, and a growing clinical literature assesses its links with borderline personality disorder [15]. Moreover, even if most ESSENCE conditions have been classified on the DSM-IV Axis I, learning disorders and ASDs have had their place on Axis II alongside with the personality disorders. Personality traits are, for instance, assumed to be normally distributed in populations, and rating scales have been developed and normalized accordingly [16, 17].

To advance our understanding of ESSENCE, the focus of the present study will be on specific developmental cognitive-emotional capacities as measured by the Temperament and Character Inventory's (TCI) [18] scales of Self-directedness and Cooperativeness. These metacognitive strategies to direct behavior are partly learned, language dependent, and serve as principles to guide executive functions [18]. In contrast to executive functions, that are trained to become automatized, Self-directedness and Cooperativeness require metacognition, that is, thinking about thinking, a “theory of mind” in relation to oneself and to others, in order to achieve the simultaneous experiencing of being a person, being with others, understanding what happens in this being, and being able to adjust behavior to constructive strategies. In adult and child psychiatry, these personality dimensions have been salient predictors of health and adaptation to adversities [19–23]. Self-directedness and Cooperativeness have also been inversely linked with ASD and ADHD in a continuous model in the normal population [24]. Self-directedness indicates how responsible, purposeful, and resourceful an individual is when it comes to achieving his or her goals and values and to identify the self as autonomous. Cooperativeness indicates how well adapted the individual is in getting along with others fairly and flexibly, combing intuition with ethical principles and to identify the self as an integral part of groups and society. Low scores have been found in personality disorders, mood disorders, and psychotic disorders. These scales have, therefore, been proposed to form an overall measure of mental health and adaptive skills, with low scores as a general marker of mental health problems [17, 25].

Based on this literature, we expected that children with different combinations of ESSENCE would consistently show low scores in Self-directedness and Cooperativeness, and that the scores would be specifically associated with dysfunctions and/or suffering in important areas (at school or home, in peer groups). If so, Self-directedness and Cooperativeness could be suggested as a dimensional global measure of the impact of the different, mostly—genetic ESSENCE symptom profiles (i.e., ADHD, ASDs, LDs, or DCD). Interventions promoting Self-directedness and Cooperativeness could reasonably be assumed to improve the individual's possibilities to cope with his or her ESSENCE disabilities (e.g., inattention, communication problems, tics, eating problems, opposition, or compulsions). It is, to the best of the authors' knowledge, unknown if different constellations of ESSENCE are associated with Self-directedness and Cooperativeness and if this can be discerned on a population-level, taking the population distribution into account.

The aim of the present study was twofold:

1. to map, continuously and categorically, ESSENCE in relation to Self-directedness and Cooperativeness and dysfunction and suffering;
2. to relate ESSENCE to Self-directedness, Cooperativeness and dysfunction and suffering on a population level.

## 2. Methods

### 2.1. Subjects

The participants in this study were recruited from the ongoing Child and Adolescent Twin Study in Sweden (CATSS). Parents of all 9-year-old twins in Sweden born from 1992 and onward (the years 1993–1995 also included 12-year-old twins) were asked to participate in a telephone interview containing the Autism-Tics ADHD and other Comorbidities inventory (A-TAC) [26, 27]. The response rate in CATSS is roughly 80% and currently comprises >22 000 twins. Parents of twins born between 1992 and onwards, where one or both twins in a pair were screened positive in the A-TAC for ADHD, ASDs, conduct disorder, oppositional defiant disorder (OCD), developmental coordination disorder and learning disabilities, plus healthy controls, were invited to participate in a follow-up study (CATSS-questionnaire). CATSS-questionnaire has answering response frequency of 60% [12] and includes, among other instruments, a parental version of the Junior-Temperament and Character Inventory (J-TCI) [28]. The sample used here consists of 2032 individuals of whom 140 were not eligible due to missing scores on the J-TCI (>5% missing responses), which rendered a final sample of 1892 (boys = 1040, girls = 852; 1121 aged 9 years old, 771 aged 12 years old). For a detailed description of CATSS, please see Anckarsater and colleagues' article [12].

### 2.2. Measures

#### 2.2.1. A-TAC

*ESSENCE.* The A-TAC is a parental telephone interview that was designed to screen for neurodevelopmental disorders; it has been validated three times cross-sectionally [26, 27, 29, 30], once longitudinally [11], and once independent of the creators research group [31]. The A-TAC consists of 96 items that are scored “1” for “yes,” “0.5” for “yes, to some extent,” and “0” for “no.” The ASDs-domain in A-TAC consists of three modules: language (6 items), social interaction (6 items), and flexibility (5 items), collapsed these modules give an ASDs-score ranging from 0 to 17. The ADHD-domain consists of two modules: concentration and attention (9 items) and impulsiveness and activity (10 items), which collapsed give an ADHD-score ranging from 0 to 19. The LDs consist of one module comprising three items (score ranging between 0 and 3). The motor control module, corresponding to DCD, consists of one item (score ranging between 0 and 1). In the present study, we used the cut-offs derived by Larson et al. [27]: for ASDs ≥ 4,5 (sensitivity .91/specificity .80), for ADHD ≥ 6.0 (.91/.73), for LDs ≥ 1.0 (.92/.60), and for DCD ≥ 0.5 (.63/.68). Distribution, heritability estimates, and Cronbach's **α** for all scales are given elsewhere in other publications [12, 27].

*Dysfunction and Suffering.* For each module, in which at least one items is scored “0.5” or “1,” the parents were asked (1) if the endorsed symptoms have led to dysfunction at school, among peers, or at home, or (2) if the child suffers from the symptoms. These questions are also scored “1” for “yes,” 0.5 for “yes, to some extent,” and “0” for “no.” A scale measuring dysfunction and suffering was created using the answers from the aforementioned seven modules in the A-TAC, and thus, theoretically, the raw score ranged from 0 to 14. Using the means and standard deviations from the full CATSS-sample (i.e., 0.26 ± 1.15), the scale was standardized by transforming the raw scores into T-scores.

#### 2.2.2. Junior-Temperament and Character Inventory (J-TCI)

The J-TCI was designed to measure temperament and character during childhood [32] according to the psychobiological model of personality [18]. In the present study, we used the parent-rated version of the J-TCI, which comprises 108 items that are answered using a binary scale (“yes” coded as 1, “no” coded as 0). Here, we focus on the Self-directedness (20 items, e.g., “My child does not blame other people or circumstances for his/her choices”) and Cooperativeness (20 items e.g., “My child treats everyone with kindness and respect no matter how unimportant or bad they are”) scales. For each scale, the raw score was transformed to T*-*scores using the means and standard deviations from the Swedish validation of the parent version of the J-TCI [28] (for Self-directedness: 16.8 ± 2.7; for Cooperativeness: 16.8 ± 2.5). Moreover, as the sum of Self-directedness and Cooperativeness is commonly used as a measure of character maturity [33], we also summarized the raw scores into a single scale (SD + CO). Using the means and standard deviations of the SD + CO composite (33.6 ± 4.4) from the Swedish validation of the J-TCI [28], the sum was then transformed to T*-*scores. In T*-*scores, 50 represents the mean and a difference of 10 from the mean indicates a difference of one standard deviation. With regard to Self-directedness and Cooperativeness, immaturity is measured as 2 standard deviations below the mean, that is, a T*-*score of 30 [33].

### 2.3. Analyses

#### 2.3.1. Continuous

The mean T-score of SD + CO, Self-directedness, and Cooperativeness were calculated for each A-TAC score. The distributions of autistic traits, ADHD-traits, LD-traits, and DCD-traits were converted into population percentiles based on the results from the baseline CATSS-study, *n* = 17 220 [12]. The mean T-scores for the SD + CO composite were then calculated for each population percentile, in order to describe the impact of ESSENCE traits on a population level. Similarly, T-scores of dysfunction and suffering were calculated for each A-TAC score and each population percentile.

#### 2.3.2. Categorical

Using the four categorical screening diagnoses, ten different categories of coexisting conditions that always included ADHD or ASDs were created taking all different constellations of coexisting conditions into account (i.e, ADHD + ASDs + DCD + LDs or ASDs + DCD, etc.). In addition, four “pure” categories were created: ASDs, ADHD, LD, or DCD only). All categories were mutually exclusive; that is, it was not possible to belong to more than one category. Mean T-scores for Self-directedness, Cooperativeness, SD + CO, and dysfunction and suffering were then calculated for all of the 14 categories.

## 3. Results

### 3.1. Continuous Measures of ESSENCE Conditions

For each increasing A-TAC scale step (i.e., one more endorsed symptom question on ADHD, ASDs, LDs, or DCD), the mean SD + CO score decreased and the number of reported dysfunctions and sufferings increased (Tables 1 and 2). This pattern was similar for all four types of ESSENCE A-TAC scores (see Tables S1 and S2 of the Supplementary Material available online on http://dx.doi.org/10.1155/2013/416981 for results regarding Self-directedness and Cooperativeness).

As compared to the ASDs score, a higher ADHD score was required to affect Self-directedness and Cooperativeness and to cause dysfunction and/or suffering (at 7 or more points in the ADHD score; mean SD + CO was lowered by more than one standard deviation, while the corresponding effect was noted at 3 points on the ASDs score). The number of reported areas of dysfunction and/or suffering increased at about the same scores.

### 3.2. Analysis of ESSENCE Groups/Categories

**Figure 1:**
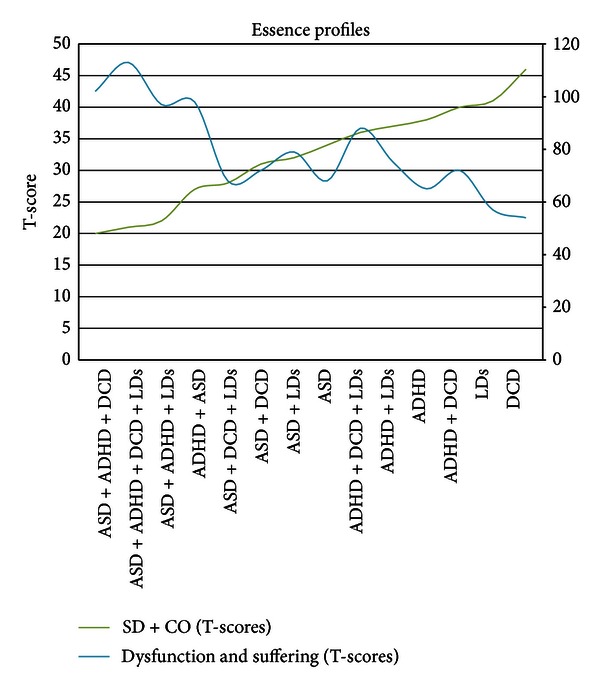
The graph shows means in SD + CO and dysfunction and suffering (T-scores) as a function of ESSENCE profiles. SD + CO: left-axis scale; dysfunction and suffering: right-axis scale.

The mean T-scores in Self-directedness, Cooperativeness, SD + CO, and dysfunction and suffering are reported for all diagnostic groups in Table 3. In Figure 1, we have plotted the mean T-scores of SD + CO and dysfunction and suffering. The presence of any ESSENCE condition was related to a decrease in Self-directedness and Cooperativeness, including DCD (i.e., motor discoordination) that is often overlooked in psychiatry. The diagnostic combinations that included ASDs resulted in Self-directedness and Cooperativeness scores at least two standard deviations below the mean. A trend could also be discerned showing that the higher the number of concomitant conditions, the greater the decrease in Self-directedness and Cooperativeness. For instance, the combination of ASDs + ADHD+ DCD + MR displayed a mean T-score of 21 while ADHD + LDs and ASDs + MR had mean scores of 37 and 32, respectively, while higher mean SD + CO T-scores could be seen in groups without any concomitant conditions (ASDs = 34; ADHD = 38; LDs = 41; DCD = 46). Again, a decrease in Self-directedness and/or Cooperativeness was accompanied by reports of dysfunction and suffering.

### 3.3. Deficits in Self-directedness and Cooperativeness and Dysfunction and Suffering in relation to the Population Distribution of ESSENCE

**Figure 2:**
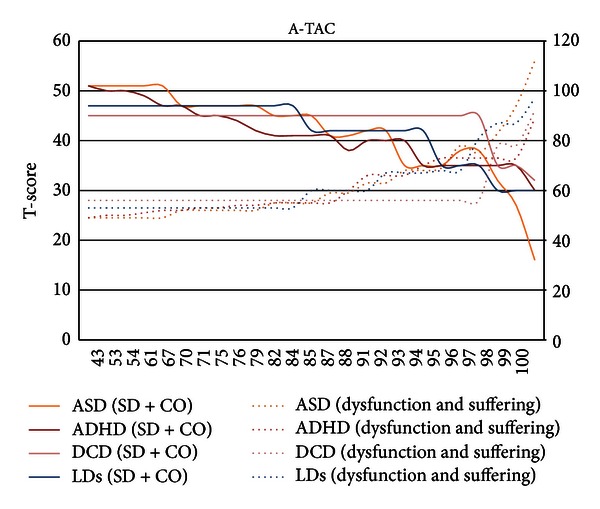
The graph shows means in SD + CO and dysfunction and suffering (T-scores) as a function of the population percentile. SD + CO: left-axis scale; dysfunction and suffering: right-axis scale.

In a subsequent step, to avoid the effects of scoring ESSENCE problems by symptom scales that contain different numbers of items and are ordinal data, the different A-TAC symptom scores were transformed into population percentiles. Mean T-scores of SD + CO and dysfunction and suffering were plotted against these population percentiles (see Figure 2). A consistent pattern of population impact across the ESSENCE conditions (DCD with is limited distribution only vaguely reflected the pattern) ensued. Going from the average towards the extreme end of the population distribution, dysfunction and suffering were first noticeable at about the 82–84th percentile of ESSENCE symptom scores, where also the SD + CO score had decreased by about one standard deviation. At about the 98 percentile in ADHD, ASDs, and LDs, a surge in dysfunction and suffering was mirrored by a rapid decrease in mean SD + CO (Figure 2). At the 99th percentile, SD + CO was 2 standard deviations below the mean, mirroring high ratings of dysfunction and suffering. The seemingly different impact of each scaling step in the A-TAC algorithms thus disappears when transformed into percentiles.

## 4. Discussion

In this paper, we forward the knowledge of the field by showing that (1) the number of symptoms of ESSENCE is continuously associated with both deficient development of Self-directedness and Cooperativeness and psychosocial dysfunction and suffering on a population level in children aged 9 or 12. (2) Combinations of ESSENCE, especially those including ASDs, were associated with particularly low scores in Self-directedness and Cooperativeness and with higher rating scores of dysfunction and suffering. (3) The impact of symptoms of ESSENCE on deficient Self-directedness and Cooperativeness and psychosocial dysfunction and suffering was found in a milder form in about 16% of all children and a severe form in about 2%, which corresponds to an underlying normal distribution of overall mental health.

Based on the findings presented here, the following conclusions may be drawn. First, not only in adults [25, 33, 34] and adolescents [35–41] but also in childhood, the Self-directedness and Cooperativeness scales measure an intrinsic aspect of global mental health. At least, low-to-very low scores identify something shared by individuals who (also) exhibit symptoms of mental disorders and associated functional deficits and/or suffering. It may be argued that low Self-directedness and Cooperativeness is merely an epiphenomenon, a “marker” of the neuropsychiatric dysfunctions, and that lacking sense of responsibility, self-control, and social skills such as tolerance to others, empathy, and the ability to be helpful and showing compassion is part of the definitions of ADHD and ASDs. The clear association to the dysfunction and suffering scale here, however, speaks against this stance. How successful a person is in influencing his or her own behavior and in interacting with others depends on sophisticated metacognition (i.e., thinking about thinking, taking different perspectives, and evaluating possible consequences of actions on others and on oneself) based on intuition and other emotions, mental strength and education-given knowledgeable insights, goals, and respect. Deficiencies in these processes, expressed as undercontrolled behavior patterns with destructive consequences, may be seen as “endophenotypes” of the ESSENCE conditions. This is supported by recent results [24], where cross-twin cross-trait correlations indicate commonalities in the etiology in ADHD, ASDs, and Self-directedness and Cooperativeness. A viable approach for future twin studies would be to disentangle the etiological association between Self-directedness and Cooperativeness and ESSENCE. The results presented here indicate that goal setting, effortful control, respect for the own person and for others (all descriptions of high Self-directedness and Cooperativeness) are important in order to avoid developing the full picture of psychosocial dysfunction and suffering associated with severe forms of ESSENCE.

Second, the results seem to indicate worse trajectories for those that are impaired in multiple ESSENCE domains, which further highlights the need for broad assessments. Further studies should investigate whether the number of conditions is an independent risk factor even when the total score is taken into account.

Third, the continuous population association results further advance the notion that there is no qualitative demarcation between traits and disorders [10, 30]. The mirroring of the SD + CO to dysfunction and suffering suggests that ESSENCE traits, or a third factor associated to both ESSENCE and SD + CO, give rise to mental health vulnerability in a considerable group of developing individuals, while the impact is severe on a small group of individuals; that is, if problems are prolonged, they may be more prone to develop deleterious disorders like schizophrenia.

### 4.1. Clinical Implications

The main focus of interventions for ESSENCE has been directed towards the core-symptoms of the disorders per se (e.g., pharmacotherapy for inattention/hyperactivity, special education for learning problems, and sociocommunicative training for ASDs). However, there is now evidence from children [24], adolescents [42], and adults [20] to state that Self-directedness and Cooperativeness are intrinsic to ADHD and ASDs, and conversely, that behavior problems referred to as personality disorders, deviant personality traits, destructive behavior patterns, or merely poor education, on the population level and in many individual cases, have antecedents in the form of childhood neuropsychiatric problems as included in the ESSENCE definition.

Recent population-based longitudinal studies show increases in Self-directedness and Cooperativeness (which is an indicator of increasing responsibility and relatedness) with age (from 20 to 45) [43]. Prospective studies show that parental care giving and home environment are more strongly associated with offspring's Self-directedness and Cooperativeness than with offspring's temperament later in adulthood [44]. Moreover, the possibility to treat deficiency in Self-directedness and Cooperativeness in relation to ESSENCE in the developing years is supported by a recent adolescent study, where the possibility to develop a sense of responsibility (i.e., self-directed behavior) and cooperation even when constrained by genetic and environmental adversity was assessed [42]. Monozygotic cotwins of probands reporting severe personality problems (i.e., extremely low in Self-directedness and Cooperativeness) were found to vary widely into the normal range. This pattern was also found among monozygotic co-twins to probands who had a parent-rated DSM-IV disruptive behaviour disorder (i.e., attention-deficit/hyperactivity disorder, oppositional defiant disorder, or conduct disorder). In other words, Self-directedness and Cooperativeness are to some degree malleable even under genetic and environmental adversity. Thus, the identification of Self-directedness and Cooperativeness as a core deficit in ESSENCE might be a promising starting point to focus on for professionals in the treatment of children impaired within multiple domains. The success of such collective effort might end in the alleviation of dysfunctions and self-related suffering.

According to Cloninger [45], therapeutic interventions focusing on the development of positive emotions and different constructs of Self-directedness and Cooperativeness (e.g., sense of responsibility and purpose, helpfulness, and empathy) have been shown to enhance well-being and provide alleviation for problems and disabilities in the general population, as well as in most, if not all, mental disorders [33, 45–50]. When compared with cognitive behavioral therapy or psychotropic medications alone, these interventions show improvements in Self-directedness and Cooperativeness and in treatment adherence among individuals with mental disorders [45]. The question whether Self-directedness and Cooperativeness can be increased among children with ESSENCE, however, remains to be formally tested in controlled trials, before personality disorders and their spectra of associated mental health problems are established in early adulthood. It is plausible to suggest that the adolescent years provide a window of opportunities for improving an individual's Self-directedness and Cooperativeness. Indeed, neuroimaging research suggests that cognitive and behavioral changes occurring during adolescence might be understood from the perspective of increased “executive functioning” (e.g., attention, response inhibition, regulation of emotion, organization, and long-range planning; for a review, see [51]).

### 4.2. Limitations

The findings in this paper should be viewed in the light of some limitations. (1) The scores for dysfunctions and suffering have not been formally validated. In support of the assumption that these questions provide valid information on children, it may be argued that they are concrete questions asked after describing every possible diagnostic symptom of the condition, and that they have convergence with the symptom scores and the scores presented here. (2) The finding that the presence of multiple ESSENCE domains was associated with a more severe impairment might have been an artifact from the rating process, as broader problems in several modules gave more opportunities to answer dysfunction and suffering questions. However, the concomitant decrease in SD + CO would speak against the notion of this as merely an artifact. In addition, it is unusual that individuals with ASDs do not report one single ADHD symptom [52], and since only one fully or partially endorsed question in any of the A-TAC modules would have led to the questions of dysfunction and suffering, it seems unreasonable that this would explain a large part of the observed effect. (3) The low sensitivity and specificity of the DCD-cutoff warrants caution when interpreting the results, at the same time it was associated (±0.5 standard deviations) with a decreased SD + CO and an increase in dysfunction and suffering.

## 5. Conclusion

Self-directness and Cooperativeness are related to a number of problems included in the ESSENCE and to dysfunction and suffering in individuals screening positive for ASDs, ADHD, LDs, and DCD. These associations can also be discerned on a population level. Based on symptom scores, it would seem that ASDs have a stronger influence, compared to that of ADHD, on Self-directedness and Cooperativeness, but this effect disappeared when the population distribution was accounted for. The results from the present study would indicate that developmental deficits in Self-directedness and Cooperativeness affect a group of about 16% of all children moderately and 2% of all children severely.

Medicalization of problems (“it was the ADHD that smashed the window” or “it's a disease, nothing he/she can help”) may contribute to the development of even lower Self-directedness and Cooperativeness and thereby even more severe mental health problems. Therapeutic interventions focusing on Self-directedness and Cooperativeness might provide a novel method for child psychiatry in its approach to ESSENCE and also provide constructive ways to acknowledge the reality of neurocognitive problems.

## Supplementary Material

The Supplementary Material shows the results of the analysis regarding Self-directedness and Cooperativeness in which for each increasing A-TAC scale step (i.e., one more endorsed symptom question on ADHD, ASDs, LDs, or DCD), the mean Self-directedness and Cooperativeness score decreases (Tables S1 and S2).

## Figures and Tables

**Table 1:** Number of individuals (*N*), means (*M; *bold typed), and standard deviations (sd) in SD + CO (T-scores) for each gate score in the A-TAC modules: ASDs, ADHD, LDs, and DCD.

A-TAC score		0	0.5	1.0	1.5	2.0	2.5	3.0	3.5	4.0	4.5	5.0	5.5	6.0	6.5	7.0	7.5	8.0	8.5	9.0	9.5	10.0	10.5	11.0	11.5	12.0	≥12.5
ASDs	*N *	657	291	215	141	121	75	69	57	35	33	22	24	9	19	18	10	11	83*								
	*M *	**50.7**	**47.0**	**44.7**	**41.3**	**42.3**	**34.8**	**35.1**	**38.4**	**37.6**	**31.8**	**32.3**	**29.9**	**30.2**	**22.2**	**21.2**	**28.9**	**30.5**	**16.0**								
	Sd	9.6	11.4	13.2	15.1	12.4	13.7	14.1	15.1	11.3	16.8	20.4	17.1	16.2	13.6	18.2	16.5	13.5	16.2								

ADHD	*N *	504	164	139	98	80	80	57	56	56	46	51	46	39	37	33	29	32	25	27	20	24	27	24	23	16	154
	*M *	**51.3**	**49.5**	**49.1**	**46.9**	**45.1**	**44.3**	**41.8**	**41.5**	**41.3**	**40.7**	**35.8**	**41.0**	**39.1**	**35.1**	**33.9**	**37.9**	**32.7**	**37.5**	**39.7**	**34.2**	**31.9**	**34.8**	**31.1**	**29.6**	**26.1**	**26.1**
	Sd	9.5	11.3	9.7	10.9	12.2	14.0	13.0	15.6	13.6	13.7	17.4	12.4	13.9	14.1	15.2	14.2	13.7	15.1	14.6	15.5	15.7	17.2	15.6	16.9	16.2	18.6

LDs	*N *	1180	203	157	106	102	55	88																			
	*M *	**46.6**	**42.0**	**41.6**	**35.5**	**35.2**	**29.7**	**29.6**																			
	Sd	13.0	15.3	15.4	16.7	18.2	18.3	16.5																			

DCD	*N *	1591	211	89																							
	*M *	**44.9**	**34.7**	**31.7**																							
	Sd	13.9	17.6	20.4																							

*≥8.5.

**Table 2:** Number of individuals (*N*), means (*M*; bold typed), and standard deviations (sd) in dysfunction and suffering (T-scores) for each gate score in the A-TAC modules: ASDS, ADHD, LDs, and DCD.

A-TAC score		0	0.5	1.0	1.5	2.0	2.5	3.0	3.5	4.0	4.5	5.0	5.5	6.0	6.5	7.0	7.5	8.0	8.5	9.0	9.5	10.0	10.5	11.0	11.5	12.0	≥12.5
ASDs	*N *	657	291	215	141	121	75	69	57	35	33	22	24	9	19	18	10	11	83*								
	*M *	**48.9**	**51.9**	**54.8**	**59.5**	**63.3**	**67.9**	**68.9**	**77.9**	**74.9**	**78.7**	**89.2**	**87.8**	**81.1**	**103.3**	**100.2**	**80.8**	**103.5**	**112.3**								
	Sd	4.0	8.5	11.5	14.0	16.1	18.7	19.3	18.9	20.9	20.0	23.4	26.6	24.5	26.5	23.7	20.2	21.7	30.4								

ADHD	*N *	504	164	139	98	80	80	57	56	56	46	51	46	39	37	33	29	32	25	27	20	24	27	24	23	16	154
	*M *	**48.8**	**49.7**	**51.0**	**52.1**	**52.8**	**54.0**	**53.9**	**55.3**	**59.0**	**60.3**	**59.9**	**63.0**	**70.5**	**66.8**	**73.7**	**67.2**	**78.4**	**74.3**	**74.8**	**78.6**	**80.3**	**75.9**	**92.1**	**87.1**	**92.6**	**99.4**
	Sd	3.7	5.1	7.3	9.3	8.0	11.2	9.9	12.1	13.6	15.2	15.3	16.6	21.4	21.8	21.9	21.4	24.5	19.8	21.7	21.2	28.2	21.3	28.1	19.8	25.3	28.8

LDs	*N *	1180	203	157	106	102	55	88																			
	*M *	**53.5**	**60.2**	**66.6**	**67.9**	**80.7**	**86.6**	**96.5**																			
	Sd	13.2	18.7	24.5	20.6	27.1	28.3	29.5																			

DCD	*N *	1591	211	89																							
	*M *	**56.5**	**78.0**	**91.8**																							
	Sd	16.1	28.9	35.0																							

*≥8.5.

**Table 3:** Number of individuals and mean (T-scores) in Self-directedness, Cooperativeness, SD + CO, and dysfunction and suffering across ESSENCE profiles.

ESSENCE profile	*N *	Self-directedness	Cooperativeness	SD + CO	Dysfunction and suffering
ASDs + ADHD + DCD	25	23	26	20	102
ASDs + ADHD + DCD + LDs	79	23	28	21	113
ASDs + ADHD + LDs	50	24	29	22	97
ADHD + ASDs	28	33	28	27	98
ASDs + DCD + LDs	9	28	35	28	68
ASDs + DCD	9	39	29	31	72
ASDs + LDs	9	30	41	32	79
ASDs	20	37	35	34	68
ADHD + DCD + LDs	44	33	44	36	88
ADHD + LDs	105	34	44	37	75
ADHD	151	39	41	38	65
ADHD + DCD	28	40	43	40	72
LDs	181	43	48	41	57
DCD	75	46	47	46	54
