# Supplementary material for: Self-Directedness and Cooperativeness, Psychosocial Dysfunction and Suffering in ESSENCE
Source: ScientificWorldJournal. 2013 Apr 28;2013:416981. doi: 10.1155/2013/416981 (PMC3655653; doi:10.1155/2013/416981)
Supplement: Supplementary file 1 — The Supplementary Material shows the results of the analysis regarding Self-directedness and Cooperativeness in which for each increasing A-TAC scale step (i.e., one more endorsed symptom question on ADHD, ASDs, LDs, or DCD), the mean Self-directedness and Cooperativeness score decreases (Tables S1 and S2). [file 416981.f1.doc]

**SUPPLEMENTAL MATERIAL TO:**

**Gubernatorial neurocognitive functions, psychosocial dysfunctioning and suffering in ESSENCE**

TABLE S1. Number of individuals (*N*), means (*M;* **bold typed**) and Standard Deviations (*Sd*.) in Self-directedness (*T-scores*) for Each Gate Score in the A-TAC Modules: ASDs, ADHD, LDs, and DCD.

| **A-TAC SCORE** |  | **0** | **0.5** | **1.0** | **1.5** | **2.0** | **2.5** | **3.0** | **3.5** | **4.0** | **4.5** | **5.0** | **5.5** | **6.0** | **6.5** | **7.0** | **7.5** | **8.0** | **8.5** | **9.0** | **9.5** | **10.0** | **10.5** | **11.0** | **11.5** | **12.0** | **≥12.5** |
| --- | --- | --- | --- | --- | --- | --- | --- | --- | --- | --- | --- | --- | --- | --- | --- | --- | --- | --- | --- | --- | --- | --- | --- | --- | --- | --- | --- |
| **ASDs** | *N* | 657 | 291 | 215 | 141 | 121 | 75 | 69 | 57 | 35 | 33 | 22 | 24 | 9 | 19 | 18 | 10 | 11 | 83* |  |  |  |  |  |  |  |  |
|  | *M* | **50.7** | **46.7** | **45.0** | **41.3** | **41.6** | **33.7** | **34.7** | **37.6** | **37.3** | **32.0** | **32.7** | **31.3** | **28.9** | **27.0** | **22.6** | **29.6** | **32.2** | **21.2** |  |  |  |  |  |  |  |  |
|  | *Sd* | 9.8 | 11.5 | 12.8 | 14.9 | 13.5 | 13.7 | 14.3 | 13.8 | 12.9 | 15.4 | 16.8 | 16.8 | 15.8 | 14.4 | 14.3 | 12.2 | 12.9 | 13.0 |  |  |  |  |  |  |  |  |
| **ADHD** | *N* | 504 | 164 | 139 | 98 | 80 | 80 | 57 | 56 | 56 | 46 | 51 | 46 | 39 | 37 | 33 | 29 | 32 | 25 | 27 | 20 | 24 | 27 | 24 | 23 | 16 | 154 |
|  | *M* | **51.4** | **49.2** | **49.4** | **47.4** | **45.8** | **44.6** | **41.5** | **42.1** | **41.9** | **39.6** | **36.9** | **40.0** | **42.1** | **36.3** | **33.1** | **38.9** | **30.5** | **35.3** | **36.6** | **34.6** | **33.6** | **34.8** | **31.8** | **26.9** | **26.2** | **27.4** |
|  | *Sd* | 9.2 | 11.1 | 9.6 | 9.8 | 12.8 | 12.9 | 13.5 | 14.4 | 14.6 | 14.4 | 16.8 | 12.7 | 10.3 | 12.9 | 13.8 | 15.8 | 12.9 | 15.3 | 15.4 | 13.0 | 15.5 | 15.6 | 14.5 | 15.1 | 13.5 | 15.6 |
| **LDs** | *N* | 1180 | 203 | 157 | 106 | 102 | 55 | 88 |  |  |  |  |  |  |  |  |  |  |  |  |  |  |  |  |  |  |  |
|  | *M* | **47.5** | **41.3** | **40.5** | **35.1** | **33.9** | **29.6** | **26.8** |  |  |  |  |  |  |  |  |  |  |  |  |  |  |  |  |  |  |  |
|  | *Sd* | 11.8 | 14.9 | 14.4 | 16.8 | 17.7 | 14.5 | 14.0 |  |  |  |  |  |  |  |  |  |  |  |  |  |  |  |  |  |  |  |
| **DCD** | *N* | 1591 | 211 | 89 |  |  |  |  |  |  |  |  |  |  |  |  |  |  |  |  |  |  |  |  |  |  |  |
|  | *M* | **45.0** | **35.0** | **32.6** |  |  |  |  |  |  |  |  |  |  |  |  |  |  |  |  |  |  |  |  |  |  |  |
|  | *Sd* | 13.6 | 16.3 | 17.1 |  |  |  |  |  |  |  |  |  |  |  |  |  |  |  |  |  |  |  |  |  |  |  |

Note: * ≥ 8.5.

TABLE S2. Number of individuals (*N*), means (*M;* **bold typed**) and Standard Deviations (*Sd*.) in Cooperativeness (*T-scores*) for Each Gate Score in the A-TAC Modules: ASDs, ADHD, LDs, and DCD.

| **A-TAC SCORE** |  | **0** | **0.5** | **1.0** | **1.5** | **2.0** | **2.5** | **3.0** | **3.5** | **4.0** | **4.5** | **5.0** | **5.5** | **6.0** | **6.5** | **7.0** | **7.5** | **8.0** | **8.5** | **9.0** | **9.5** | **10.0** | **10.5** | **11.0** | **11.5** | **12.0** | **≥12.5** |
| --- | --- | --- | --- | --- | --- | --- | --- | --- | --- | --- | --- | --- | --- | --- | --- | --- | --- | --- | --- | --- | --- | --- | --- | --- | --- | --- | --- |
| **ASDs** | *N* | 657 | 291 | 215 | 141 | 121 | 75 | 69 | 57 | 35 | 33 | 22 | 24 | 9 | 19 | 18 | 10 | 11 | 83* |  |  |  |  |  |  |  |  |
|  | *M* | **50.4** | **48.2** | **46.0** | **44.0** | **45.4** | **40.9** | **40.3** | **42.9** | **41.9** | **37.5** | **37.5** | **34.8** | **37.9** | **26.0** | **29.0** | **34.8** | **34.8** | **21.4** |  |  |  |  |  |  |  |  |
|  | *Sd* | 9.4 | 10.8 | 13.2 | 14.1 | 12.5 | 14.9 | 15.0 | 15.5 | 10.9 | 17.5 | 24.0 | 16.1 | 16.7 | 14.3 | 20.3 | 20.8 | 17.9 | 18.5 |  |  |  |  |  |  |  |  |
| **ADHD** | *N* | 504 | 164 | 139 | 98 | 80 | 80 | 57 | 56 | 56 | 46 | 51 | 46 | 39 | 37 | 33 | 29 | 32 | 25 | 27 | 20 | 24 | 27 | 24 | 23 | 16 | 154 |
|  | *M* | **50.7** | **49.9** | **49.2** | **47.4** | **46.0** | **45.8** | **44.8** | **43.5** | **43.3** | **44.9** | **39.2** | **44.9** | **39.4** | **38.5** | **39.9** | **40.7** | **40.7** | **43.9** | **46.4** | **38.8** | **35.8** | **39.7** | **36.5** | **39.0** | **33.6** | **32.3** |
|  | *Sd* | 9.5 | 11.4 | 10.1 | 11.2 | 11.5 | 14.5 | 13.7 | 16.2 | 12.8 | 13.2 | 17.0 | 12.2 | 16.4 | 15.9 | 15.6 | 14.4 | 16.2 | 14.8 | 13.0 | 18.2 | 16.4 | 17.2 | 16.7 | 17.2 | 18.7 | 19.3 |
| **LDs** | *N* | 1180 | 203 | 157 | 106 | 102 | 55 | 88 |  |  |  |  |  |  |  |  |  |  |  |  |  |  |  |  |  |  |  |
|  | *M* | **46.6** | **45.4** | **45.4** | **40.5** | **41.4** | **36.3** | **39.2** |  |  |  |  |  |  |  |  |  |  |  |  |  |  |  |  |  |  |  |
|  | *Sd* | 13.0 | 14.1 | 15.2 | 15.5 | 16.5 | 20.4 | 18.1 |  |  |  |  |  |  |  |  |  |  |  |  |  |  |  |  |  |  |  |
| **DCD** | *N* | 1591 | 211 | 89 |  |  |  |  |  |  |  |  |  |  |  |  |  |  |  |  |  |  |  |  |  |  |  |
|  | *M* | **46.4** | **39.3** | **36.6** |  |  |  |  |  |  |  |  |  |  |  |  |  |  |  |  |  |  |  |  |  |  |  |
|  | *Sd* | 13.2 | 17.3 | 20.3 |  |  |  |  |  |  |  |  |  |  |  |  |  |  |  |  |  |  |  |  |  |  |  |

Note: * ≥ 8.5.
